# Working from home and its challenges for transformational and health-oriented leadership

**DOI:** 10.3389/fpsyg.2022.1017316

**Published:** 2022-12-01

**Authors:** Dorothee Caroline Tautz, Katharina Schübbe, Jörg Felfe

**Affiliations:** Department of Work, Organizational, and Business Psychology, Helmut Schmidt University, Hamburg, Germany

**Keywords:** working from home, virtual leadership, transformational leadership, health-oriented leadership, virtual communication

## Abstract

The Covid-19 crisis forced many employees to abruptly relocate their workplace from the office to their homes. As working from home is expected to remain part of our working world, consequences for leadership need to be examined. Our study aims to investigate the concrete challenges regarding the feasibility of transformational leadership and health-oriented leadership in this remote setting. Therefore, we collected quantitative and qualitative data of 23 leaders and 18 employees from various organizations in Germany. Both groups were asked to report their experiences during working from home in comparison to the traditional office setting. Findings of our study provide a comprehensive understanding regarding the underlying mechanism that impede transformational and health-oriented leadership in the remote setting. Among them participants reported a lack of social presence, limited informal chats, communication difficulties and lack of mutual trust. Based on our findings we derive practical implications for leaders and HR practitioners.

## Introduction

The COVID-19 crisis provoked immense changes in the world of working. To avoid getting infected and spreading the virus many employees were forced to abruptly relocate their workplace from the office to their homes (Kaushik and Guleria, 2020). Accordingly, working from home (WFH) increased significantly in most organizations (Hans-Böckler-Stiftung, 2021) and brought both challenges and opportunities. Positive consequences that emerged with WFH are for example better integration of family and work, less distraction from colleagues and less commuting (Al-Habaibeh et al., 2021) whereas reduced communication with colleagues, isolation and an inadequate home office environment (e.g., no separate room for work activities, poor internet connection…) have come up as challenges (Xiao et al., 2021). One group that experiences a particularly strong transformation and increased challenges in their daily working life are leaders (Kirchner et al., 2021). Previous literature has already claimed that the principles and concepts of leadership found in the traditional office setting cannot be simply transferred to the remote setting (Hoch and Kozlowski, 2014). For example, leaders report an increase in working hours, additional administration and difficulties in keeping in touch with their followers (Kirchner et al., 2021). Another challenge that goes along with the reduced contact are difficulties in motivating followers and in maintaining trust in their work ethics and engagement (Avolio et al., 2001; Bell and Kozlowski, 2002). While there is already some understanding of general challenges and demands for leadership, the impact of WFH for specific leadership styles is still unclear. In our study we focus on the two well-established leadership styles transformational leadership (TFL; Bass and Riggio, 2006) and health-oriented leadership (HOL; Franke et al., 2014) as their effectiveness for employees’ performance (Wang et al., 2011) and health (Franke and Felfe, 2011; Arnold and Rigotti, 2021; Kaluza et al., 2021) was proven in numerous studies for the traditional office setting. Leaders who execute these leadership styles are perceived as charismatic and inspiring (Bass and Riggio, 2006) and are well aware of the health status of their employees (Franke et al., 2014). This leads to increased performance, commitment and satisfaction among employees (Judge and Piccolo, 2004; Braun et al., 2013; Klebe et al., 2021). However, as social interaction is limited during WFH (Lal et al., 2021) especially these employee-oriented leadership styles that thrive on regular contact and face-to-face communication might suffer.

Previous studies on the effectiveness of remote transformational leadership provide inconsistent results. While Purvanova and Bono (2009) report an increase of TFL in an experimental remote setting, others found that effectiveness decreased with geographically dispersed teams (Hoch and Kozlowski, 2014; Eisenberg et al., 2019). Reasons for these contradictory results could be the feasibility of TFL and how leaders behave in the remote context. Therefore, it is necessary to investigate the challenges regarding the feasibility of TFL and HOL in the WFH context and to derive practical implications. To date there are only few studies that have addressed this question. Among them, Liebermann et al. (2021) conducted interviews with leaders in the public sector to investigate the difficulties to display TFL when switching from the office to WFH and identified general demanding working conditions (e.g., workload, time pressure, role conflicts) which become stronger in the remote context. Similarly, Efimov et al. (2020) conducted interviews to analyze HOL behaviors of remote leaders and found that distance makes it more difficult to detect signs of stress.

As literature is scarce, there is a need for a more comprehensive understanding of how specific characteristics of the digital context impair or facilitate the feasibility of sub-dimensions of TFL and HOL. To close this research gap, we collected quantitative and qualitative data from 23 leaders and 18 employees who were asked to directly compare their experiences during WFH with the office setting. We chose a quantitative and qualitative approach because the combination of both methods provides a deeper insight than either method alone (Bryman, 2003). While the quantitative data allow us to identify systematic differences between WFH and working in the office, the qualitative data shed deeper light on the reasons and causes for the differences between the two contexts. Further we decided to collect data from both leaders and employees as past research has only focused on the leader perspective (Efimov et al., 2020; Liebermann et al., 2021). We decided to include the perspectives of employees as they are the ones who are directly affected by the leadership styles and may report different experiences.

The purpose of our study is to bring new insights into the factors that might impede TFL and HOL during WFH. Based on our findings we derive practical implications for leaders. Leaders need to be aware of the challenges for leadership during WFH and the factors that influence feasibility. Only by addressing these challenges, leaders will be able to successfully and effectively lead their employees in the remote context. These implications are especially relevant when leaders and employees spend most of their working time at home and the possibility to compensate the challenges by meeting regularly in the office is limited. Our findings will contribute to the literature on WFH with focusing on TFL and HOL by identifying relevant boundary conditions and offering new perspectives for further research.

## Theory and research questions

### Leadership and working from home

The Covid-19 crisis acted as an accelerator for WFH (Wethal et al., 2022). Before, in most organizations only small parts of the staff worked regularly from home, often with agreements that allow only one working day from home in a week. The COVID-19 pandemic interrupted this situation and enforced all employees to work full time from home if their job could be accomplished outside of the office (Steude, 2021). Many employees benefited from WHF due to more flexible working hours and better integration of work and private life (Al-Habaibeh et al., 2021). Because of that about 50% of the employees wish to remain in a hybrid working model in the future with 2 or 3 working days a week from home and even 21% wish to spend almost their entire working time from home (Krick et al., 2022). Also, many organizations support WFH and tend to permanently transfer some of their employees to remote positions to save costs (Gartner, 2020). As it is to be expected that WFH remains part of our working world, consequences and challenges need to be examined and addressed.

One group that experiences particular challenges are leaders (Kirchner et al., 2021). Their leadership role becomes more challenging. At the same time it gets more relevant to keep the team together and to ensure cooperation among followers (Contreras et al., 2020). Leadership tasks can be generally divided into two categories: (1) monitoring and managing of ongoing activities in the department and within the team and (2) communication, collaboration and shaping team processes (Bell and Kozlowski, 2002). Regarding the first leadership function, leaders have additional administrative tasks like reorganizing projects and ensuring collaboration during WFH. Regarding the second leadership function, leaders suffer from limited communication and interaction with their followers (Kirchner et al., 2021). The digitalized communication might provoke misunderstandings because the tone of a message is not conveyed in written language. For example, humor and irony are more difficult to understand (van Wart et al., 2019). Moreover, for leaders it is more difficult to access their followers due to the physical distance. It needs more effort to start a conversation and especially spontaneous informal chats are limited (van Wart et al., 2016; Kirchner et al., 2021). Leaders need to make an extra effort to create cohesion and team spirit between followers and to develop new employees into one work unit in the remote setting (Kozlowski et al., 1996). Overall, it is important to understand that leadership in the remote context follows its own rules (Avolio and Kahai, 2003). Leaders must adapt to the new remote conditions and adopt new communication and relationship building methods (Contreras et al., 2020).

While there is already some understanding regarding general challenges for leadership in the new employees into one work unit in the remote setting (Kozlowski et al., 1996), literature regarding the consequences of WFH for specific leadership styles that depend on regular communication and face-to-face interaction is scarce. Two of these leadership styles which are well-established are TFL (Bass and Riggio, 2006) and HOL (Franke and Felfe, 2011; Franke et al., 2014). In the following we will elaborate on specific challenges and consequence for these leadership styles during WFH and explore if and to what extent they are still feasible in a setting with limited interaction and communication.

### Transformational leadership

TFL is one of the most studied leadership styles in the current literature and has proven its effectiveness for performance, job satisfaction and commitment in the traditional office setting in numerous studies (Judge and Piccolo, 2004; Tims et al., 2011; Wang et al., 2011; Braun et al., 2013). The concept of TFL aims to increase intrinsic motivation of employees and differentiates four sub-dimensions: (1) idealized influence (acting as a role model; transmitting values and beliefs), (2) inspirational motivation (inspiring, motivating with demanding goals; emphasizing team spirit), (3) intellectual stimulation (encouraging followers to think outside the box) and (4) individualized consideration (knowing and considering the individual needs and strengths of followers; Bass and Riggio, 2006).

Empirical evidence found in the traditional office setting cannot be simply transferred to the remote setting (Hoch and Kozlowski, 2014). Effectiveness and feasibility might differ in a context with limited interaction, digital communication and lack of social interaction. However, the few studies that have investigated TFL in the remote context show inconsistent results. While some studies found that the effectiveness decreases (Hoch and Kozlowski, 2014; Eisenberg et al., 2019), others report the opposite (Purvanova and Bono, 2009). Possible reasons for a decrease of TFL could be the lack of contact and the use of digital media for communication (Hoch and Kozlowski, 2014; Eisenberg et al., 2019). Eisenberg et al. (2019) speculate that leaders’ authenticity is declined due to the distance and lack of face-to-face interaction. In contrast, Purvanova and Bono (2009) found that TFL is more effective for team performance in a setting with only e-mail communication, compared to a face-to-face setting. They explain their results by suggesting that leaders might put more effort into displaying TFL behaviors in the remote setting to compensate the uncertain and ambiguous situation. In a current study, Liebermann et al. (2021) conducted interviews in the public sector during the Covid-19 crisis and found that primarily demanding working conditions become stronger in the WFH context and therefore challenge the feasibility of TFL.

In the following we outline which specific challenges may influence the feasibility of the different sub-dimensions of TFL during WFH. *Idealized influence* might be hindered because of the limited contact and interaction between leaders and employees. They only talk occasionally with each other or not even at all (Kirchner et al., 2021). This lack of contact might impede being perceived as role model. As the bonding between leader and employee is looser and more fragile, inspiring messages from the leader might be perceived as inauthentic and out of place. Further missing information in conversations like tone, mimics, gesture and body language might lead to misunderstandings (Wang et al., 2020) and hence impair the effects of inspirational messages. This is in line with Eisenberg et al. (2019) who speculate that the geographic distance makes it more difficult for leaders to be perceived as authentic role models and to reach followers on an emotional level. A challenge for *inspirational motivation* is primarily that leaders no longer receive much information from their followers. Bell and Kozlowski (2002) assume that in the remote setting it is difficult for leaders to capture the atmosphere within the team and to manage team dynamics. Therefore, it might be more difficult to share a common vision and to encourage the team spirit. Also, Liebermann et al. (2021) found that the lack of communication impairs the assessment of the followers’ level of motivation so that leaders do not know when to intervene. *Intellectual stimulation* might be challenged due to the fact that there is often no adequate technological equipment like videoconferencing tools (Liebermann et al., 2021) so that leaders and followers cannot elaborate on ideas face-to-face with each other and have no possibility to share their screens to show something and ensure common understanding. Also, meetings are more efficient and more accurately timed so there might be less room for brainstorming, letting thoughts flow and taking time to develop ideas. Creative thinking also needs breaks. But these might feel strange and lead to misunderstandings during digital communication because the other person does not know if the break is related to a technical problem, distraction or thinking processes. Further, the generation of new ideas often happens in spontaneous chats (McAlpine, 2018) which are limited during WFH. *Individualized consideration* may decrease in the remote context as this dimension particularly thrives on regular contact and communication. To consider the individual needs and strengths of followers, leaders must know them very well. However, during WFH leaders do barely get any private information about their followers as spontaneous informal chats are limited. Eisenberg et al. (2019) postulate that as it is more difficult for leaders to recognize when followers need help and support, they feel more inhibited to approach them proactively.

Research Question 1: Which challenges do leaders perceive in executing (a) idealized influence, (b) inspirational motivation, (c) intellectual stimulation, and (d) individualized consideration when WFH compared to working in the office?

Research Question 2: To what extent do employees perceive (a) idealized influence, (b) inspirational motivation, (c) intellectual stimulation, and (d) individualized consideration from their leader when WFH compared to working in the office? And what are the reasons for different perceptions?

### Health-oriented leadership

While TFL rather focuses on increasing employees’ performance, satisfaction, and engagement, HOL specifically aims to increase health and well-being (Franke et al., 2014). As a multidimensional construct, HOL consists of the three components StaffCare (leaders considering and actively promoting their followers’ health), leader SelfCare and follower SelfCare (taking care of one’s own health). Each of these components can be broken down into the three sub-dimensions of value, awareness, and behavior (Franke et al., 2014). In our study we mainly focus on StaffCare and its three sub-dimensions because we aim to particularly investigate the challenges for leadership. SelfCare we conducted just in employees’ interviews. StaffCare *value* means attaching importance and willingness to take over responsibility for the health of followers as a leader. *Awareness* means being aware and sensitive of warning signals regarding the health status of followers. *Behavior* refers to promoting and engaging in concrete health-oriented activities (e.g., encouraging to take breaks and participate in occupational health programs, improving work environments). While there is much evidence for positive effects of HOL for employees’ health in the traditional office setting (Franke et al., 2014; Klug et al., 2019; Arnold and Rigotti, 2021; Kaluza et al., 2021) it is unclear if these findings can be transferred to the remote setting. To date, we are only aware of one study that deals with HOL in a remote setting. Efimov et al. (2020) conducted an interview study with leaders of virtual teams and identified first insights regarding feasibility and possible action steps to promote HOL. However, the concrete challenges and opportunities regarding the three sub-dimensions are still unclear. In the following we will outline which specific challenges may influence the feasibility of the different sub-dimensions. Regarding *value*, it can be assumed that leaders attach less importance to promoting followers’ health in the WFH context because they have less access to their followers and therefore, they might feel less responsible for their health. In their study, Efimov et al. (2020) found that leaders do not feel responsible for employees’ health but for creating a healthy environment. However, it is questionable if leaders succeed in doing this. Moreover, as communication is very limited (Kirchner et al., 2021) leaders might use the few conversations they have with their followers for rather talking about tasks and goals and not about health issues. *Awareness* might also be challenged during WFH. Warning signals for health issues are often conveyed over mimics, body language and tone. This nonverbal information is especially limited when communication only happens *via* digital media (Fayard et al., 2021). So, it can be expected that it becomes more difficult for leaders to recognize when their followers feel stressed or sick. This assumption goes along with the findings from Efimov et al. (2020). Additionally, followers might be more hindered to disclose private health issues to their leader because it is more difficult to develop a trustful atmosphere in the remote context. In terms of the third sub-dimension health-oriented *behavior*, we assume that leaders see fewer possibilities to influence working conditions from the distance. For example they do not see the working hours of their employees, how often they take breaks or if they suffer from technical challenges. Based on this, leaders might take less action steps to proactively promote followers’ health.

Research Question 3: Which challenges do leaders perceive in executing the three StaffCare dimensions (a) value, (b) awareness, and (c) behavior when WFH compared to working in the office?

Research Question 4: To what extent do employees perceive StaffCare and its three dimensions (a) value, (b) awareness, and (c) behavior when WFH compared to working in the office? And what are the reasons for different perceptions?

## Materials and methods

To answer our research questions, we collected quantitative data with a standardized survey and qualitative data with semi-structured interviews from 23 leaders and 18 followers who are employed in different organizations in Germany.

### Sample

All participants were recruited through personal networks of the authors. Inclusion criteria were leaders and employees who regularly WFH and from the office and are therefore able to compare both settings. Among the participants with leadership responsibility were 10 women and 13 men at the age between 29 and 62 years (*M* = 42.02). They are all employed and work in one of the following industries: IT, consulting, public sector, engineering, event management, automobile, or retail. At the time of the data collection, they had between 1 and 30 years of leadership experience (*M* = 7.62) and worked between 1 and 5 days a week from home (*M* = 2.71). Among the participants without leadership responsibility were 13 women and 5 men at the age between 20 and 57 years (*M* = 39.12). They are also all employed, report to a direct leader and work in one of the following industries: IT, consulting, public sector, finances, insurances, e-commerce, logistics or retail. At the time of the data collection, they worked between 1 and 4 days a week from home (*M* = 2.50).

### Procedure

We started the data collection with a quick warm-up. In this phase, we collected descriptive data from the participants and informed them about the purpose of the study and data security. Afterwards participants received a survey with items regarding the four dimensions of TFL and the three dimensions of HOL and were asked to rate them on a scale from 1 (does not apply at all) – 5 (fully applies). For each dimension they received one item and were asked to first rate to what extent it applies when they are working at home and second to rate to what extent it applies when they are working collocated in the office. Leaders were asked to do a self-assessment of their own leadership style and employees were asked to assess the leadership style of their direct leader. In a next step, the qualitative interview started. Participants were asked to elaborate on their ratings. For each item they explained why they perceive it as challenged in the WFH context compared to the traditional office setting. Or, when their rating was higher in the WFH context which opportunities they experienced. The interview ended with a closing statement and gave the participants the opportunity to report any experiences that they had made and were not addressed to this point.

### Materials

The items used to assess TFL were derived from the MLQ (Felfe, 2006) while the items to assess HOL are based on the instrument of Franke et al. (2014). The interview guide was first pre-tested with academics and practitioners to check for its content validity and comprehensibility. The modified interview guideline was then pilot tested with two participants to check its appropriateness for the target population before the interview process started. The interviews were conducted between June 2021 and July 2022 *via* videocall or telephone and lasted between 45 and 90 min.

### Analysis

The qualitative data were analyzed and interpreted according to Mayring (2022). The interviews were read and screened for common patterns and similarities by the authors. In a first step, categories were developed deductively based on the previous literature. Then the coding tree was enriched inductively with categories based on the transcripts. All steps of the analysis were carried out by the authors. Agreements and disagreements of the screenings and categorization from the authors were discussed and resulted in adjusting the categories for finding the best fit of the data. To analyze the quantitative data, we calculated means and paired t-tests. The premises for the paired t-test were met except for the premise of normal distribution. However, due to the explorative approach of our study and since the paired t-test is considered to be very robust to violations (Pagano, 2012), we decided to continue with the data.

## Results

### Transformational leadership

#### Idealized influence

Leaders perceive restriction to display idealized influence in the remote setting compared to the traditional office setting [WFH: *M* = 3.59, *SD* = 0.96; office: *M* = 4.45, *SD* = 0.51; *t*(21) = 4.31; *p* = 0.000]. Employees also perceive less idealized influence when working remotely although the difference is not significant [WFH: *M* = 3.11, *SD* = 1.23; office: *M* = 3.50, *SD* = 1.15; *t*(17) = 1.94; *p* = 0.069].

The main reason for the decrease of idealized influence mentioned by the leaders is the lack of contact and social presence. Leaders report that role modelling needs face-to-face interaction and goes hand in hand with perceiving the leader throughout the day and seeing how they work and interact with people *(“Functioning as role model is created through presence and face-to-face interaction. Working from home is a barrier to this”)*. However, there are also voices claiming that authenticity increases because followers see their leaders in a more private manner, e.g., when children interrupt a meeting *(“Followers perceive me in a private setting. It happens that my son comes in during videoconferences. This makes me more accessible”)*. Further, leaders mentioned that online meetings are rather task-oriented. There is no room to talk about values and beliefs as it is in the office during face-to-face meetings *(“Online meetings are more efficient and task-oriented. There is barely room for talking about values, beliefs and visions”)*. However, leaders report that the challenges depend on the relationship they have with their followers. While less challenges occur with followers they know very well, more occur with new followers to whom they do not have a strong bonding. The interviewed employees confirm that the lack of social presence is a main challenge *(“When I work at home, I hear almost nothing from my manager. That’s why he does not influence me or conveys any values and beliefs”)*. They also reported that that their leaders do not trust them to work efficiently or at all at home which impairs their relationship in terms of mutual confidence and makes it difficult to perceive leaders as role models *(“My manager has no trust in me and my colleagues. She does not think we are really working when we are at home”)*.

It can be concluded that idealized influence deteriorates in a setting with limited contact and social presence. Perceiving the leader throughout the day and in different situations is important for idealized influence as well as opportunities to talk about private, non-work-related topics. It seems that strong relationships and trust between leaders and followers may compensate the challenges. An overview of the challenges and opportunities is provided in Table 1.

**Table 1:** Challenges and opportunities for idealized influence.

		*M*	*SD*	Challenges (−) and Opportunities (+)
Leaders *t*(21) = 4.31; *p* = 0.000)	WFH	3.59	0.96	– **lack of social presence impedes role modelling** “Functioning as role model is created through presence and face-to-face interaction. Working from home is a barrier to this.” - **task-oriented online meetings impede vision communication** “Online meetings are more efficient and task-oriented. There is barely room for talking about values, beliefs and visions.” + **visibility of privacy supports authenticity** “Team members perceive me in a private setting. It happens that my son comes in during videoconferences. This makes me more accessible.”
	Office	4.45	0.51	+ **social presence supports role modelling** “I think in the office I rather act as role model because my team members experience me at my work throughout the whole day and I constantly interact with them.”
Employees *t*(17) = 1.94; *p* = 0.069)	WFH	3.11	1.23	– **lack of social presence impedes communication of values** “When I work at home, I hear almost nothing from my manager. That’s why he does not influence me or conveys any values and beliefs.” - **lack of contact reduces trust and confidence** “My manager has no trust in me and my colleagues. She does not think we are really working when we are at home.”
	Office	3.50	1.15	+ **social presence supports role modelling** “In the office, I see how my manager works, talks to people and manages things. This inspires me a lot.” + **spontaneous, informal chats support communication of values** “I often speak spontaneously or during breaks with my manager. In these conversations I learn a lot about his values and beliefs.”

#### Inspirational motivation

Leaders perceive more restrictions to display inspirational motivation during WFH compared to the office setting [WFH: *M* = 3.59, *SD* = 1.05; office: *M* = 4.32, *SD* = 0.78; *t*(21) = 3.46; *p* = 0.002] and also employees perceive less inspirational motivation at home [WFH: *M* = 3.00, *SD* = 1.19; office: *M* = 3.50, *SD* = 1.30; *t*(17) = 2.67; *p* = 0.015].

For leaders, the lack of social presence and interaction make it difficult to capture the team atmosphere. Leaders do not receive non-verbal cues, so it is difficult to assess the true emotions of their followers and to react appropriately. In contrast to the office setting, they do not get a feeling of the team spirit or upcoming conflicts *(“In the remote setting it is difficult to sense when conflicts are upcoming or when the general mood decreases.”)*. Further, digital communication and especially asynchronous communication hamper the transfer of enthusiasm and motivation. Even during videoconferences, the leaders receive barely any stimulating feedback on what they said so it is difficult and exhausting for them to reach their followers on an emotional level *(“I do not know how to transfer enthusiasm and motivation via e-mail or chat. And also, during videoconferences it’s difficult because I do not get any non-verbal signals.”)*. The interviewed employees also report that lack of contact and social presence are main issues. From the distance, employees would not call their leaders to talk about motivation, team spirit and further topics that go beyond the actual work *(“I would not dare to call my leader to talk about my level of motivation or the atmosphere in the team. I know that he is very busy and I do not want to interrupt him with something that is not task-related.”)*. They also confirm that the communication *via* digital technologies hampers the transfer of enthusiasm. In the office they also get non-verbal cues from their leader which are important to convey emotions *(“Usually my manager is someone who is very good at conveying motivation and enthusiasm for our long-term goals. But when we work from home, he does not do it at all. Or I just do not perceive it.”)*.

Overall inspirational motivation seems to decrease in the remote setting (see Table 2). Main reasons for this are the lack of social presence and the communication *via* digital tools which makes it almost impossible to convey enthusiasm and motivation as important non-verbal cues are missing. Also, for leaders it is more difficult to sense the team spirit and to intervene when conflicts appear.

**Table 2:** Challenges and opportunities for inspirational motivation.

		*M*	*SD*	Challenges (−) and Opportunities (+)
Leaders *t*(21) = 3.46; *p* = 0.002)	WFH	3.59	1.05	– **lack of contact (feedback) impedes capturing the team atmosphere** “In the remote setting it is difficult to sense when conflicts are upcoming or when the general mood decreases.” **- digital communication hampers transfer of enthusiasm** “I do not know how to transfer enthusiasm and motivation *via* e-mail or chat. And also, during videoconferences it’s difficult because I get any non-verbal signals.”
	Office	4.32	0.78	**+ contact (feedback) supports capturing the team atmosphere** “In the office I see the emotions and level of motivation of my team members. So, I can easily intervene when something is going on” **+ spontaneous, informal chats transmit enthusiasm** “Motivating my team members usually happens in spontaneous informal chats and not during discussion of tasks. These chats happen very often when we are both in the office.”
Employees *t*(17) = 2.67; *p* = 0.015	WFH	3.00	1.19	– **lack of contact impedes motivating the team** “I would not dare to call my leader to talk about my level of motivation or the atmosphere in the team. I know that he is very busy and I do not want to interrupt him with something that is not task related.” - **digital communication hampers transfer of enthusiasm** “Usually my manager is someone who is very good at conveying motivation and enthusiasm for our long-term goals. But when we work from home, he does not do it at all. Or I just do not perceive it.”
	Office	3.50	1.30	+ **social presence supports motivating the team** “In the office I communicate regularly with my leader so it easier to talk about motivation issues or issues within the team.”

#### Intellectual stimulation

The quantitative data reveal that leaders find it more challenging to intellectual stimulate their followers when they are at home compared to the office setting [WFH: *M* = 3.68, *SD* = 1.09; office: *M* = 4.09, *SD* = 0.87; *t*(20) = 1.56; *p* = 0.134]. Employees rated the intellectual stimulation in both situations identical [WFH: *M* = 4.18, *SD* = 0.88; office: *M* = 4.18, *SD* = 1.02].

Although the differences of ratings are smaller and not significant there are still some challenges that leaders report. For example, they mentioned that they face difficulties to identify problems and challenges of their followers due to the lack of contact. As followers ask less questions and barely approach their leaders, it is difficult to know when problems need to be solved *(“It’s difficult for me to know if my followers have problems or to what extent they make progress with their tasks.”)*. On the other side leaders described that followers are more autonomous which stimulates their own problem solving. They are more asked to develop solutions on their own, re-consider former working patterns and find new ways which increase their competencies and skills *(“My followers are more on their own. They are more asked to find solutions and re-consider their working patterns by themselves.”)*. A further challenge is that common creative thinking processes are impeded in online meetings without face-to-face communication and less possibilities for spontaneous visualization *(“What is missing is the opportunity to go to the blackboard together and develop something new.”)*. Cooperation in online conferences is also more difficult because there is no real eye contact and leaders hardly get feedback from followers to assess if they have a common understanding *(“It is hard to get spontaneous reactions in web meetings.”)* and if followers are still mentally present and think along *(“I know that during online meetings my followers often do other things simultaneously on their computer as I do sometimes. This makes it harder to discuss.”)*. Online meetings are often shorter and more on point so that there is less room for brainstorming or developing ideas together. Without the eye contact it is more difficult to endure conversation breaks *(“The pauses are unpleasant so that it goes on quickly instead of reflecting in silence.”)*. This is even worsened when technical problems appear and the connection breaks down regularly *(“When there are technical problems during online meetings a lot from the energy gets lost and followers rather hold back and do not say anything at all.”)*. Employees rated the intellectual stimulation equally between WFH and in the office. However, they confirmed that they are less likely to approach their leader with questions in the remote setting *(“My manager is less reachable. I feel more inhibited to call and ask questions compared to the office where we are always in direct contact.”)*. On the other side, they report that they enjoy having more autonomy and being asked to develop ideas and solutions by themselves *(“When working from home I have a lot more freedom. I tend to make my own decisions instead of constantly asking my leader for approval.”)*.

Intellectual stimulation seems to be the dimension that is the least affected by the remote context. However, it depends on the kind of issues that are discussed. While talking about task-related questions that are relatively easy to answer works equally well in the remote setting, particularly brainstorming and creative thinking processes to develop new strategies or new working patterns are impeded. Reasons are the restrictions of digital communication, the lack of social presence and technical problems. An overview can be found in Table 3.

**Table 3:** Challenges and opportunities for intellectual stimulation.

		*M*	*SD*	Challenges (−) and Opportunities (+)
Leaders *t*(20) = 1.56; *p* = 0.134)	WFH	3.68	1.09	– **lack of contact impedes identification of problems** “It’s difficult for me to know if my team members have problems or to what extent they make progress with their tasks.” - **digital communication impairs creative thinking processes** “What is missing is the opportunity to go to the blackboard together and develop something new.” **+ less contact supports autonomy and interdependence of team members** “My team members are more on their own. They are more asked to find solutions and re-consider their working patterns by themselves.”
	Office	4.09	0.81	+ **face-to-face interactions with direct feedback support creativity** “Creative thinking processes like brainstorming is easier in the office because I get direct feedback from my team members.”
Employees *no mean difference*	WFH	4.18	0.88	– **lack of contact hampers asking questions** “My manager is less reachable. I feel more inhibited to call and ask questions compared to the office where we are always in direct contact.” **+ less contact supports autonomy and interdependence** “When working from home I have a lot more freedom. I tend to make my own decisions instead of constantly asking my leader for approval.”
	Office	4.18	1.02	**+ social presence makes it easier to ask questions** “In the office, I can always ask my leader about anything, because he is in the office right next to me.”

#### Individualized consideration

Leaders find it easier to individually consider their employees in the office compared to WFH [WFH: *M* = 3.55, *SD* = 1.01.; office: *M* = 4.32, *SD* = 0.57; *t*(21) = 3.93; *p* = 0.001]. Employees report the same whereas here differences are not significant [WHF: *M* = 3.56, *SD* = 1.20; office: *M* = 3.67, *SD* = 1.03; *t*(17) = 1.46; *p* = 0.163].

Leaders report that for knowing and considering their followers, they need regular informal, non-work-related chats. Informal conversation about private matters usually happens during common breaks or other spontaneous interactions. However, the lack of contact in the remote setting makes these interactions very scarce *(“For me it is difficult to consider the needs because I do not know them. When we work from home, informal chats to talk about private, non-work-related issues hardly ever take place.”)*. Further, it is more difficult to detect if a follower is unsatisfied or unhappy because non-verbal cues like facial expressions, tone or mood are missing. Leaders also feel that the communication over digital media impedes the willingness of their followers to disclose private matters *(“When my followers work from home, they actually never talk with me about private issues or emotional things. I think it’s strange for them to do this in an e-mail or over the phone.”)*. Especially asynchronous media like e-mail or chat inhibit the communication of personal issues. Also, during synchronous meetings over telephone or video, the communication is rather task-oriented and there is less room for sharing non-work-related information.

Employees confirm that informal chats become very limited so that there are fewer opportunities to talk about personal issues *(“When I work from home, I do not have informal chats with my leader. We barely speak directly at all and when we do it is completely task-related.”)*. Accordingly, they claim that they often do not feel recognized by their leader. They have the feeling that their leaders do not care about them when they are at home as leaders do not know what they are working on or how they are doing *(“I do not have the feeling that my manager knows what I’m doing and what my needs are.”)*.

Our results show that individualized consideration decreases during WFH due to the lack of regular contact and informal spontaneous communication (see Table 4). While leaders think their followers are less willing to reach out to them and reveal private information, their employees have the feeling that their leaders lose interest in them as geographic distance increases. The communication over digital media even impedes the situation because non-verbal cues are missing and meetings become more formal and task-oriented.

**Table 4:** Challenges and opportunities for individual consideration.

		*M*	*SD*	Challenges (−) and Opportunities (+)
Leaders *t*(21) = 3.93; *p* = 0.001	WFH	3.55	1.01	– **lack of informal communication impedes consideration of needs** “For me it is difficult to consider their needs because I do not know them. When we work from home, informal chats to talk about private, non-work-related issues hardly ever take place.” - **distance and digital communication impair disclosure of employees** “When my team members work from home, they actually never talk with me about private issues or emotional things. I think it’s strange for them to do this in an e-mail or over the phone.”
	Office	4.32	0.57	**+ richer communication supports consideration of needs** “In the office, I sense how my team members are really doing because I see their facial expressions and body language.” **+ frequent communication supports consideration of needs** “I am constantly in exchange with my team members and spend every break with them. So, I get a very good impression of the current needs.”
Employees *t*(17) = 1.46; *p* = 0.163	WFH	3.56	1.20	– **lack of informal communication impedes consideration of needs** “When I work from home I do not have informal chats with my leader. We barely speak directly at all and when we do it’s completely task-related.” - **distance and digital communication support feeling of not being recognized** “I do not have the feeling that my manager knows what I’m doing and what my needs are.”
	Office	3.67	1.03	+ **frequent communication supports being recognized** “In the office, I we regularly communicate with each other. My leader gives me the feeling that she is interested in how I am doing and what my needs are.”

### Health-oriented leadership

#### Value

The quantitative ratings show that leaders rate followers’ physical and psychological health promotion less during WFH compared to the office setting [WFH: *M* = 3.18, *SD* = 1.40; office: *M* = 4.09, *SD* = 0.87; *t*(21) = 3.46; *p* = 0.002].

In the interviews they state that they feel clearly less responsible for their follower’s health during WFH. Hence, they rate the importance of health lower (“*As a leader, it is very important to me that our work environment is beneficial to our health. However, I can implement it much better in the office, perhaps because I feel more responsible.”)*. As a reason leaders mentioned that their possibilities of influencing their followers’ working environment at home is limited (*“I do not see how my employees work at home and therefore cannot influence it.”)*. Instead, they ask for more individual initiative from followers (*“As a leader, you are familiar with the risks that occur in the office for your own followers. But when working from home, you do not know the personal living conditions and so you cannot influence them. Employees have a higher responsibility for themselves.”)*. Followers confirm that their leaders value employees’ health less during WFH compared to working in the office [WHF: *M* = 3.06, office: *M* = 3.81; *t*(17) = 3.12; *p* = 0.006]. Employees feel that the importance of health is better emphasized in an office context (*“I have the feeling that my leader can better demonstrate the value of health in the office.”)*. During WFH, employees even perceive that leaders attach more importance on the fulfilment of work tasks and their performance than on their health (*“In the daily work routine at home, my leader seems to place task assignments above the importance of our health.”)*. Because of that employees feel more self-responsible for their own health (*“I think it is difficult for my supervisor to be responsible for my health during working from home. I see more of the responsibility on myself.”)*.

Overall, both leaders and employees perceive a decrease of health-oriented value because leaders have less possibilities to influence the working environment at home (see Table 5). Both groups think that responsibility for health rather shifts from the leader to the followers compared to the office setting.

**Table 5:** Challenges and opportunities for Value – HoL.

		*M*	*SD*	Challenges (−) and Opportunities (+)
Leaders *t*(21) = 3.46; *p* = 0.002	WFH	3.18	1.40	– **lower responsibility due to less control and influence** “I do not see how my employees work at home and therefore cannot influence it.”
	Office	4.09	0.87	+ **higher responsibility** “I can implement it much better in the office, perhaps because I feel more responsible.”
Employees *t*(17) = 3.12; *p* = 0.006	WFH	3.22	1.48	– **increased self-responsibility** “I think it is difficult for my supervisor to be responsible for my health during working from home, even if it is important to him. I see more of the responsibility on myself.” - **priority of task assignment** “In the daily work routine at home, my leader seems to place task assignments above the importance of our health.”
	Office	3.89	1.13	+ **higher visibility** “I have the feeling that my leader can better demonstrate the value of health in the office.”

#### Awareness

From the leaders’ perspective there is considerably less awareness for their followers’ health when WFH compared to working in the office [WFH: *M* = 2.86, *SD* = 0.94; office: *M* = 4.23, *SD* = 0.87; *t*(21) = 5.43; *p* = 0.000].

Leaders reported that there are less opportunities for interaction and poorer communication quality with their followers during WFH. This means also less time for being aware for the mental and physiological health of their followers (*“I barely see my employees and therefore it’s difficult to know their current concerns.”)*. This contrasts with the situation in the office, where spontaneous and informal conversations often take place. Leaders can recognize inconsistencies and signs for psychological stress in followers’ behavior when working in the office (*“Mental health warning signs are easier to detect when you are constantly crossing each other and do not need specific scheduled conversations.”)*. From leaders’ perspective it is unclear whether recognizing warning signals works equally well *via* video conferencing. Some report that it makes little or no difference. Others, however, say they miss the non-verbal cues (*“I am unsure if you can have the same awareness through digital communication media. It might also result from a lack of gestures and facial expressions.”)*. In addition, leaders mentioned that it is easier to deal with health concerns when they communicate face-to-face (*“Personal issues are not so easy to address during working from home. It is more pleasant in the office when you can see each other and also perceive the body language.”)*. Moreover, in the office it seems to be easier for employees to disclose concerns to their leader (*“However, I have the feeling that there is a lack of trust in digital conversations. I think my followers can open up to me better face-to-face.”)*. A further difficulty can be found in the general knowledge about health risks and its promotion. Leaders understand general health risks in the office context (*“I know the health risks for my employees that come along in our job working in the office.”)* while they do not feel sufficiently informed about the situation during WFH. Information materials and trainings regarding possible health risks often relate to the traditional office context but not to WFH. Even if it is provided, it usually contains only advice regarding better ergonomic working conditions and not to possible psychological stressors as well as health warning signals (*“I certainly do not know many psychological risk factors of my employees’ health specifically in the working from home context. There could be more than the blurring of private and working life because of too many working hours. But we did not receive any information regarding it.”)*. Similar to leaders, employees also believe that awareness of their leaders decreases during WFH [WFH: *M* = 2.17, *SD* = 1.25; office: *M* = 2.78, *SD* = 1.17; *t*(17) = 3.05; *p* = 0.007]. As a main reason for the decrease of awareness they mentioned the lack of contact and social presence (“*There is almost no contact when working from home, so my leader does not notice anything.”)*. Employees also reported that for awareness informal communication is needed. They would not talk about health issues during official meetings. In the office these topics usually arise during spontaneous informal chats or common breaks. However, these opportunities are very limited so that leaders cannot know how they feel (*“When my leader contacts me, it’s usually work-related. We barely ever talk about private matters. For example, he even does not ask how I’m doing today.”)*. Further they argued that due to digital communication leaders do not perceive any non-verbal cues like tone, facial expression or gesture. But it is precisely these cues that provide important information about how someone is doing *(“I mostly communicate via e-mail or chat with my leader. So, she does not perceive any nonverbal information from me and therefore, she cannot assess how I am doing.”)*. This leads to an additional issue, when employees feel barriers in their disclosure and do not have the confidence to address personal concerns to their leader (*“When I work from home, I do not dare to approach my leader with my private concerns and open up. The feeling of an open door is somehow missing over there.”*).

Summing up, the main reasons for the decrease of awareness during WFH are that leaders and their followers have less contact and social interaction. Important non-verbal cues are missing to assess the health and well-being of followers. Leaders also claim that they have lower competencies of health risk detection in this digital setting. An overview of the opportunities and challenges for awareness are displayed in Table 6.

**Table 6:** Challenges and opportunities for Awareness – HoL.

		*M*	*SD*	Challenges (−) and Opportunities (+)
Leaders *t*(21) = 5.43; *p* = 0.000	WFH	2.86	0.94	– **lack of information about psychological stressors** “I certainly do not know many psychological risk factors of my employees’ health specifically in the working from home context.” - **less interaction-time and contact** “I barely see my employees and therefore it’s difficult to know their current concerns.” - **insecurity regarding the use of digital communication tools** “I am unsure if you can have the same awareness through digital communication media. It might also result from a lack of gestures and facial expressions.”
	Office	4.23	0.87	**+ more trust** “I have the feeling that there is a lack of trust in digital conversations. I think my team members can open up to me better face-to-face.” + **easier to detect non-verbal warning signals** “Mental health warning signs are easier to detect when you are constantly crossing each other and do not need specific scheduled conversations.” + **personal issues are more pleasant to discuss** “Personal issues are not so easy to address during working from home. It is more pleasant in the office when you can see each other and also perceive the body language.”
Employees *t*(17) = 3.05; *p* = 0.007	WFH	2.17	1.25	– **less digital communication and interaction** “There is almost no contact when working from home, so my leader does not notice anything.” - **communication is rather task-related** “When my leader contacts me, it *via* email or telephone, and it is typically work-related. For example, he even does not ask how I’m doing today.” **- experience disclosure barriers** “When I work from home, I do not dare to approach my leader with my private concerns and open up. The feeling of an open door is somehow missing over there.”
	Office	2.78	1.17	+ **more room for personal concerns and possibilities to express them** “I feel more visible to my leader in the office, also because we cross paths, I can just walk in and, we talk more often.”

#### Behavior

From a leaders’ perspective there is less health-oriented behavior during WFH than in the office [WFH: *M* = 3.36, *SD* = 0.95; office: *M* = 3.86, *SD* = 0.71; *t*(21) = 2.32; *p* = 0.031].

Beside the challenges, leaders also report opportunities. Followers can benefit from more flexibility regarding their working conditions when WFH. They have more autonomy to organize their work in the way that suits them best. Leaders can also reduce the demands on employees with families by ensuring that they have greater autonomy in their work schedules (*“A big advantage when working from home is that it means more flexibility and freedom. This allows breaks to be taken more individually.”)* This enables their followers to develop health-promoting working conditions at home. But apart from this, for leaders there are no further instruments regarding health-promotion during WFH. They feel that their options to proactively promote health when their followers work at home are very limited (“*I cannot change the working conditions at my followers’ homes or control them in their way of working. I have no power over this at all.”)*. Employees also perceive less health-oriented behavior from their leaders when they are at home [WFH: *M* = 2.28, *SD* = 1.07; office: *M* = 2.50, *SD* = 0.99; *t*(17) = 1.72; *p* = 0.104] although the difference is not significant. Employees report that their leaders do not try to proactively take care of their psychological strains and health (*“So far, I have not received a lot of support from my leader. She does not proactively check in with me or pass any health offers. But this may also be because there are no programs in our organization.”)*.

Health-oriented behavior from leaders seems to suffer in the remote context. Although leaders have the possibilities to offer their followers more autonomy and flexibility regarding work schedules, they still feel that they have no influence when the team is at home. Employees report that they feel less supported (see Table 7).

**Table 7:** Challenges and opportunities for Behaviour - HoL.

		*M*	*SD*	Challenges (−) and Opportunities (+)
Leaders *t*(21) = 2.32; *p* = 0.031	WFH	3.36	0.95	– **no practical instruments for reducing stress** “I feel left alone. There are no specific instruments for reducing stress when my employees work from home.” - **no control or influence** “In any case, I cannot change the working conditions at my team members’ homes or control them in their way of working. I have no power over this at all.” + **offering followers more autonomy** “A big advantage when working from home is that it can mean more flexibility and freedom. This allows breaks to be taken more individually.”
	Office	3.86	0.71	+ **more control and direct influence** “I am able to provide more appropriate working hours and break schedules in the office.”
Employees *t*(17) = 1.72; *p* = 0.104	WFH	2.28	1.07	– **leaders are less proactive** “So far, I have not received a lot of support from my supervisor. She does not proactively check in with me or pass any health offers. But this may also be because there are no programs.”
	Office	2.50	0.99	**+ organizations offer health programmes** “My company provides health programs that can be participated in at the office.”

In addition to the previous findings, we also asked employees to rate their own SelfCare during WFH. Employees rated the importance of their own health promotion *(value)* less when WFH compared to working from the office [WFH: *M* = 3.78, *SD* = 0.81; office: *M* = 4.11, *SD* = 0.83; *t*(17) = 2.06; *p* = 0.055]. However, it can be noted that employees are more aware and sensitive of warning signals *(awareness)* regarding their own health status when WFH compared to the office setting [WFH: *M* = 3.61, *SD* = 0.98; office: *M* = 3.17, *SD* = 0.86; *t*(17) = −1.92; *p* = 0.072]. They also behave more health-oriented during WFH compared to working in the office [WHF: *M* = 3.83, *SD* = 1.04; office: *M* = 3.44, *SD* = 0.78; *t*(17) = −1.44; *p* = 0.168]. They find it easier to demonstrate a health-oriented behavior because on one hand they are more flexible and have more freedom to organize their working day and on the other hand they are less under the observation of their leader and colleagues (“*It’s much easier when I work from home. Here, I can rather decide for myself and I am unobserved.”*).

## Discussion

In our study we investigated challenges to display the dimensions of TFL and HOL in the remote setting by collecting quantitative and qualitative data from leaders and employees. Both groups reported quite similar differences between WFH and working in the office and both showed that feasibility of TFL and HOL seems to be considerably more difficult when WFH. We were able to identify five common core challenges that are causes for the decrease of the investigated leadership styles.

### Lack of social presence and interaction

One of the main challenges regarding the feasibility of TFL and HOL is the lack of social presence and regular interaction. Since both leadership styles are built on frequent contact and communication they suffer from a setting with limited contact. Especially non-verbal cues like facial expressions, gesture, body language and tone are very limited when communication happens only over digital media (Kayworth and Leidner, 2000; Wang et al., 2020). In our study leaders reported that this impairs the awareness for health-related warning signals as these are often not directly disclosed but can be rather discovered over non-verbal clues (e.g., when employees are unusually quiet or when they look tired and exhausted). Dimoff and Kelloway (2019) found that for most leaders the recognition of health-related warning signals is already difficult in the traditional office setting. Accordingly, it is even worse in a setting with no non-verbal cues. Moreover, in line with the findings of Hinds and Weisband (2003) we found that conveying emotions like enthusiasm and sensing the atmosphere within the team becomes increasingly difficult in the remote setting. As Avolio et al. (2001) claimed, leaders need to take over a more proactive role to ensure and create social bondings between followers. This is particularly relevant for inspirational motivation. Also idealized influence is challenged due to the lack of social presence which impairs role modelling. Employees need to perceive the leader throughout the workday and during different situations like managing tasks or interacting with others to be perceived as a role model which is limited during WFH.

### Lack of spontaneous and informal conversations

In line with Kirchner et al. (2021) we found that a key challenge to both TFL and HOL are fewer possibilities for spontaneous informal chats about private, non-work-related subjects. These seem to diminish with advancing digitization (Antoni and Syrek, 2017). In addition, meetings and discussion in the remote context become rather task-oriented and efficient. These findings are supported by Klebe et al. (2021), who confirm that task-focused communication increases within teams that face volatile and new work situations. While in the office there are many opportunities to chat about private matters apart from official meetings (e.g., encounters at the coffee machine, common lunch breaks or spontaneous chats on the floor), they do only exist to a very limited amount during WFH and need to be proactively initiated. During our interviews employees revealed that they rather disclose personal needs, emotions and health-oriented issues in exact these informal settings. Accordingly, leaders can barely recognize them during WFH which impedes particularly individualized consideration and health-oriented awareness.

### Digital communication and technical problems

In addition to the lack of social presence and informal conversation, the communication over digital media and technical problems (e.g., connection break downs, issues with software and updates) further challenge the feasibility of TFL and HOL. Leaders face especially difficulties when communication happens mainly over asynchronous media like e-mail or chat. According to the media richness theory (Daft and Lengel, 1986) these communication technologies do not include rich information like non-verbal cues and might rather lead to misunderstandings. In our study leaders reported that they find it difficult to display intellectual stimulation and develop creative thinking processes in a digital setting where they barely receive any feedback from followers in the form of eye contact, nodding or smiling. It might also happen that followers are thrown out of chats, that voices are distorted or that messages come in delated due to network problems (Kayworth and Leidner, 2000). These technical problems hinder a stable and rich conversation and might inhibit followers to proactively participate in discussions. Hogg and Reid (2006) confirm that the restrictions based on the digitalized communication make it difficult to socially connect, to communicate ideas and novel information and to assess if there is a common understanding. These and further technical problems therefore harm TFL and HOL.

### Less trust and bonding

During WFH the interpersonal relationships and ties between leaders and employees also affect TFL and HOL. Both leaders and employees feel less close to each other due to fewer interactions, less informal conversation, and increased task-orientation. A further reason for the detachment of leaders and followers is the lack of mutual trust and confidence during WFH. As some leaders do not trust their followers to work efficiently or at all from home, their bonding and perception of the leader as role model is impeded. Wang et al. (2020) confirm that in the remote setting it is difficult to assess the mood and emotions of others which hampers the formation of a strong bonding. As a strong relationship is also necessary for health disclosure in the workplace (Li and Lee, 2021), awareness decreases. It is worth highlighting, that leaders report that these experienced challenges regarding TFL and HOL apply stronger to new employees. If a strong tie and connection does already exist, challenges like lack of contact and informal discussions do not matter as much for the investigated leadership styles. Whereas regarding new employees, leaders feel more challenged and insecure to communicate effectively and convey motivation and enthusiasm. Purvanova and Bono (2009) support this finding by claiming that in the remote context leader need to increase their effort to create a strong bonding and relationship while in the office this evolves almost automatically by itself.

### Less responsibility of the leader

During WHF leaders experience that their influence on followers and control diminishes as there are fewer opportunities for monitoring (Kayworth and Leidner, 2000; Bell and Kozlowski, 2002). Therefore, leaders feel less responsible for their employees which affects particularly the HOL dimension value and behavior. Leaders stated that they cannot influence the working conditions or health behavior of their followers at home anyway. Hence, they shift the responsibility to their followers. They are rather asked to take care of themselves. Also, when challenges occur during work employees tend to feel left alone because their leader is less accessible. On the other side, the shift of responsibilities increases autonomy and independence of followers which supports the development of new competencies. They receive more opportunities to solve problems and make decisions on their own. The fact that personal responsibility and independence of employees grow goes along with previous literature suggesting that new leadership concepts like team leadership and self-leadership become more relevant in the remote setting (Kayworth and Leidner, 2000; Bell and Kozlowski, 2002; Müller and Niessen, 2018).

## Strengths and limitations

Our study provides several strengths and limitations. As an immense rise of flexible working environments and WFH is to be expected, research about its consequences for specific leadership styles are needed. Our study provides a comprehensive understanding regarding the feasibility of TFL and HOL dimensions during WFH.

However, generalizability of our findings is limited. While the sample size is adequate for interviews and our explorative approach, it is considered to be too small for quantitative hypothesis testing. Our quantitative data were not normally distributed and although the paired t-test is known to be relatively robust against violations of normal distribution, we recommend an extension of this study with a clear quantitative focus and larger sample size. Additionally, we only collected data from German employees. Also, participants without managerial responsibility were mainly female which might have led to different results than a sample with balanced gender ratio. Future studies need to validate our findings with cross-sectional or longitudinal studies with larger and more representative sample sizes. Second, the retrospective data design allowed us to investigate individual experiences and differences between WFH and working in the office. But there is a forgetfulness bias as participants may not remember all relevant factors. Also, causal claims cannot be made. Therefore, future studies need to enhance our findings with experimental settings to identify the causal effects of for example informal communication, lack of non-verbal cues or willingness to disclose on HOL and TFL. Moreover, the interviewed leaders and employees did not work together in dyads, so we did not have matched data. Due to organizational reasons, it was not possible for us to collect this kind of data. It would be interesting to compare the perceptions of leaders with the perceptions of their direct followers. We recommend for future research to consider using matched data for further qualitative or quantitative studies.

However, our study has also some strengths. One of them is that we used a mixed methods approach and collected quantitative and qualitative data to gain a comprehensive understanding. Another strength lies in our sample. As we interviewed participants within our personal network there was a huge willingness to trust and open up about their individual challenges. Further, we included data from both employees and leaders and reached a sufficiently large sample. Since both groups independently reported similar challenges, the validation of our study can be considered good. In addition, we asked the same individuals to directly compare the situation in the office with the situation at home. This with-in design allows us to identify direct differences and reasons why the feasibility of HOL and TFL is compromised in a remote setting.

## Practical implications

In our study we identified different challenges for the feasibility of TFL and HOL which must be addressed by leaders and HR practitioners to ensure effective leadership during WFH. A main challenge for TFL and HOL is the lack of contact and communication and missing of non-verbal cues. In line with Bell and Kozlowski (2002) we recommend using videoconferences over meetings without camera for team meetings and discussions of complex tasks. Benefits are that the camera transmits some non-verbal cues like facial expressions and encourages followers to be more present and proactive. Seeing the manager directly simplifies the communication of emotions such as enthusiasm and team spirit, and at the same time it makes it easier to recognize health problems. However, also in videoconferences challenges for TFL and HOL occur. Organizations need to ensure that leaders and followers have adequate equipment at home to reduce technical problems. Further, leaders need to be aware that inspirational messages might not reach their followers as expected. They might also have less possibilities to perceive health issues or other problems of followers. Therefore, they need to schedule more time during the day to actively discuss questions, needs and concerns with their followers. During these meetings leaders should be open and transparent about their own current responsibilities, tasks and challenges. Doing this might compensate the lack of social presence and facilitate perceiving the leader as role model and hence increase idealized influence. In addition, next to these formal meetings, we recommend leaders to actively take time for informal chats (e.g., virtual coffee break, virtual lunch break, team-building activities) during regular working time. Scheduling frequent interactions was also suggested by Krebs et al. (2006) to develop trust in the digital context. However, individual circumstances must be considered. It is counterproductive to intensify work–family conflicts of employees or disregard their boundaries between work and private life by arranging additional events outside of working hours (Wang et al., 2021). Leaders need to balance the amount of informal activities with their employees.

Leaders also need new feedback tools and instruments to proactive enquire and assess their follower’s concerns and the mood within the team. This allows them to intervene quickly when challenges and issues within the teams arise. Moreover, leaders need to be aware that they still have an influence on their followers and cannot just shift their responsibility towards them. Specific health-oriented instruments might increase health-related behaviors of leaders. Among our interviewed leaders, none of them received training or specific information on how to effectively lead during WFH. Accordingly, it seems that to date there is a lack of specific instruments to train leaders on promoting health-oriented behaviors (e.g., physical activity of their employees, prevention of stress and boundary management) and on detecting health-related warning signals for mental health issues.

## Conclusion

Our research was novel in exploring specific challenges for leaders regarding the feasibility of TFL and HOL during WHF. By using quantitative and qualitative data from both leaders and employees, we identified various challenges for the investigated leadership styles. Among them lack of social presence, communication difficulties, lack of mutual trust and weaker ties were reported. Our study provides a first comprehensive understanding regarding the underlying mechanism that effect TFL and HOL. Based on our findings we derived recommendations and practical implications for leaders and HR practitioners.

## Data availability statement

The raw data supporting the conclusions of this article will be made available by the authors, without undue reservation.

## Ethics statement

Ethical review and approval were not required for the study on human participants in accordance with the local legislation and institutional requirements. The patients/participants provided their written informed consent to participate in this study.

## Author contributions

DT, KS, and JF developed the research question and study design. DT and KS collected and analyzed the data and wrote the first draft of the manuscript. JF revised the manuscript. All authors contributed to the article and approved the submitted version.

## Funding

This research was funded by dtec.bw for the project: “Digital Leadership and Health.”

## Conflict of interest

The authors declare that the research was conducted in the absence of any commercial or financial relationships that could be construed as a potential conflict of interest.

## Publisher’s note

All claims expressed in this article are solely those of the authors and do not necessarily represent those of their affiliated organizations, or those of the publisher, the editors and the reviewers. Any product that may be evaluated in this article, or claim that may be made by its manufacturer, is not guaranteed or endorsed by the publisher.
